# The interface between consumers and their food environment in Myanmar: an exploratory mixed-methods study

**DOI:** 10.1017/S1368980018003427

**Published:** 2018-12-18

**Authors:** Shauna M Downs, Sara Glass, Kay Khine Linn, Jessica Fanzo

**Affiliations:** 1 Department of Health Systems and Policy, Rutgers School of Public Health, 112 Paterson Street, New Brunswick, NJ 08901 USA; 2 Berman Institute of Bioethics, Johns Hopkins University, Baltimore, MD, USA; 3 HelpAge International Myanmar, USA; 4 School of Advanced International Studies, Johns Hopkins University, Baltimore, MD, USA

**Keywords:** Food environment, Nutrition transition, Dietary patterns, Food preferences, Myanmar, Low- and middle-income countries

## Abstract

**Objective:**

To examine consumers’ perceptions of their food environments, their food consumption patterns and preferences, and to better understand the attributes of foods that are available within food environments in Myanmar.

**Design:**

An exploratory mixed-methods study using a combination of focus group discussions, market and consumer surveys.

**Setting:**

Four study settings in Myanmar were included: an upper-income township of Yangon; a lower-income township of Yangon; a middle-income township in the southern Myanmar town of Dawei; and a lower-income village in the country’s dry zone of Magway.

**Participants:**

Thirty-two women participated in the focus groups discussions, twenty market surveys were conducted and 362 consumers (both men and women) completed food consumption surveys.

**Results:**

Focus group participants indicated that the availability of a diverse range of foods had increased over time, while the quality of foods had decreased. Health was seen primarily through the lens of food safety and there was an overall lack of knowledge about which foods were more or less healthy. Consumers preferred fruits, vegetables and red meat compared with highly processed snack foods/beverages. Although consumers reported low intakes of highly processed snack foods, Burmese street food was consumed in high quantities. The market surveys suggested that fresh, minimally processed and highly processed foods were available at all markets across the study settings.

**Conclusions:**

Consumers are exposed to a variety of foods, of varying quality, within their food environments in Myanmar. Interventions aimed at increasing consumer knowledge regarding healthy diets and improving food safety are needed.

Rapid urbanization in many low- and middle-income countries (LMIC) has coincided with a nutrition transition^(^ ^1^ ^,^ ^2^ ^)^. As low-income populations migrate from rural areas to urban settings, their dietary patterns tend to shift from traditional diets (high in staples, pulses, vegetables, etc.) to those containing higher quantities of animal-sourced foods, sugar, oils, and highly processed foods and beverages (e.g. biscuits, soda, etc.)^(^ ^1^ ^)^. A corollary to these changes has been an epidemiological transition. Although a high prevalence of undernutrition persists in many LMIC, overweight and obesity and diet-related non-communicable diseases have increased, with many countries concomitantly tackling multiple burdens of malnutrition^(^ ^3^ ^)^.

Given that poor diets are a major risk factor for both undernutrition and overweight/obesity, improving food choices is essential to addressing these burdens, particularly as populations shift from rural to urban settings and face markedly different food environments^(^ ^4^ ^)^. The High Level Panel of Experts on Food and Nutrition Security describes the food environment as consisting of four components that shape food purchasing decisions: (i) availability and physical access (proximity); (ii) economic access (affordability); (iii) promotion, advertising and information; and (iv) food quality and safety^(^ ^5^ ^)^. Consumers then interface with the food environment to make decisions related to where and what foods to acquire, prepare, cook, store and eat^(^ ^5^ ^)^. These decisions have implications for their diets as well as their nutrition and health outcomes^(^ ^5^ ^)^.

The majority of the research examining food environments has been conducted in high-income countries^(^ ^6^ ^)^. This research has used a variety of methods, including mapping via geographic information systems, to examine how physical proximity to food outlets contributes to obesity and non-communicable diseases^(^ ^7^ ^)^. In the past, little attention was given to the food environments in LMIC but there has been an increased interest in capturing food environments in these contexts in recent years^(^ ^6^ ^,^ ^8^ ^,^ ^9^ ^)^, particularly as the prevalence of overweight/obesity and diet-related non-communicable diseases rise in these countries^(^ ^2^ ^,^ ^10^ ^)^. However, very little primary research has been conducted to examine food environments in LMIC. Moreover, their dynamic and informal nature creates obstacles for measuring them.

The present paper explores the food environment in the South-East Asian country of the Republic of the Union of Myanmar. Myanmar, which is situated between the borders of India, Bangladesh, China, Laos and Thailand, has suffered over 60 years of conflict, something it continues to grapple with to this day. In 2010, political reforms began in the country leading to a shift away from a military regime, with a democratic election. These political reforms have helped foster economic development in the country. Although Myanmar is one of the region’s most impoverished countries, it is considered one of the fastest growing economies in East Asia^(^ ^11^ ^)^. Growth in gross domestic product has been predicted to average 7·3 % per year from 2018 to 2022^(^ ^12^ ^)^, and the number of middle-class consumers in Myanmar is projected to double by 2020^(^ ^13^ ^)^. Alongside the country’s economic reforms, there has been a rise in foreign direct investment in Myanmar’s food and beverage sector^(^ ^14^ ^)^ and increased sales of packaged and processed foods^(^ ^15^ ^)^.

Globally, income growth is correlated with changes in dietary consumption patterns that have an impact on human health^(^ ^16^ ^)^. Alongside Myanmar’s recent economic growth, the country has begun contending with the multiple burdens of malnutrition. In 2008, the prevalence of overweight was 24 % and the prevalence of obesity was 6 % in Myanmar^(^ ^17^ ^)^. A more recent study conducted in 2015–2016 reported that 28 % of Burmese adult women were overweight and 13 % were obese, while 14 % were underweight^(^ ^18^ ^)^. Among women 30–49 years of age, about 50 % were overweight or obese^(^ ^18^ ^)^. At the same time, 29 % of children under 5 years of age were stunted in 2015–2016 and 7 % were wasted, while approximately 30 % of women of reproductive age were anaemic^(^ ^19^ ^)^. Some of these changes are likely attributable to the changing Burmese food environments and their influence on diets. Between 2000 and 2013, the availability of energy in the food supply increased from 7991 to 10 757 kJ/capita per d (1910 to 2571 kcal/capita per d), while the fat supply increased from about 38 to 69 g/capita per d over this same time period^(^ ^20^ ^)^. These changes are consistent with other countries in the region that have seen dramatic changes in the foods available within their food environments over time^(^ ^21^ ^)^.

The overarching objective of the present exploratory mixed-methods study was to characterize the food environments in four distinct settings in Myanmar. More specifically, our study aims were to: (i) examine consumers’ perceptions of their food environment and how it has changed over time; (ii) gain insight into current food consumption patterns and food preferences; and (iii) better understand the attributes of foods that are available within food environments.

## Methods

In four settings in Myanmar, a combination of focus group discussions, as well as market and consumer surveys, was conducted to characterize the food environments and how consumers interface with them to make decisions about food choices.

### Study settings

The four study settings examined were: (i) an upper-income urban township of Yangon; (ii) a lower-income urban township of Yangon; (iii) a middle-income urban township in the southern Myanmar town of Dawei; and (iv) a lower-income rural village in the country’s dry zone of Magway. The study locations were purposively selected to represent a diverse range of food environments. Yangon is the largest city in Myanmar and its former capital. Located in the lower part of the country near the Gulf of Martaban, it has a population of over 7·3 million accounting for 14·3 % of the total Burmese population and where urban upper-income and low-income townships reside simultaneously^(^ ^22^ ^)^. Dawei is in the south-eastern region of Tanintharyi and is a coastal town with a relatively small population of 125 239^(^ ^22^ ^)^. The region shares a border with Thailand and is sparsely populated. Magway is situated in the central part of the country with a population of approximately 3·9 million^(^ ^22^ ^)^. It is the main region producing edible oils in the country as well as petroleum. Data collection was conducted between June and August 2017.

### Focus groups

One focus group, combined with social mapping, was conducted in each of the four study settings. Focus group participants were purposively selected in each study setting by our local partners, in conjunction with community leaders. Participants were selected to ensure that the focus groups contained participants with a range of ages, levels of education and types of employment. The focus group participants lived within a defined area in each of the focus group settings to ensure they had access to the same markets. A semi-structured focus group guide that included questions related to food purchasing and consumption, food quality and preferences, and how food environments have changed over time was used to lead the discussions. Each focus group contained eight women participants (age range 21–61 years; mean 40 years), of a similar socio-economic status, who made food purchasing decisions in their household. Women with household incomes of 25 lakhs (∼$US 16 000) or higher were considered high-income, 10–20 lakhs ($US 6300–13 000) were defined as middle-income and women with household incomes of <10 lakhs were described as low-income. We purposefully selected participants from different socio-economic status groups to provide us with a range of perspectives in terms of food preferences and food purchasing habits, given that it is an important determinant of the foods people consume and the way in which they interface with their food environment. Focus group participants received an incentive for participation (20 000 Burmese Kyat; equivalent to approximately $US 15). A trained Burmese facilitator guided the focus group discussions. In addition to the focus group facilitator, an additional researcher took detailed notes of the focus group discussions. The focus group discussions ranged from 1·5 to 2 h to complete and were audio-recorded, transcribed verbatim and translated into English to facilitate data analysis.

Part of the focus group discussion included participatory social mapping, an ethnographic technique in which focus group participants draw maps of their local community^(^ ^23^ ^)^. The focus of the social mapping was on the food environment and it entailed noting all the places in which participants acquire food, including markets, vendors, etc. Participants were given a large white sheet of paper and were asked to draw, together as a group, the features of their local food environment (e.g. the various places where they purchase food). After mapping the food outlets that they are exposed to within their food environment, they were asked what types of foods they purchase at each of the locations. The mapping, along with the focus group discussions, helped to identify the main markets to target for conducting the market and consumer surveys. It also helped to characterize the food environments in the different study settings.

### Market surveys

The markets that focus group participants most commonly mentioned purchasing foods from were selected for the market surveys. A total of twenty market surveys were completed (five in each study setting). The market surveys consisted of information on overall market infrastructure and a checklist for commonly consumed foods within each of the following food groups: grains; pulses, nuts and seeds; dairy; meat, poultry and fish; eggs; dark leafy greens; vitamin A-rich fruits and vegetables; other vegetables; and other fruits. These food groups were selected to represent a diverse range of food items based on a validated dietary diversity indicator^(^ ^24^ ^)^. Local partners helped to identify the key local foods commonly consumed within each of the dietary diversity food groups to develop the market survey checklist. In addition, highly processed food categories, namely potato chips, instant noodles, and sugary and energy drinks, were included. For the purposes of the present paper, a subset of the foods included in the market surveys is reported. Trained enumerators conducted the market surveys after first conducting a pilot of the survey tool to identify discrepancies in the interpretation of its questions among enumerators. These discrepancies were addressed prior to collecting the study data. However, we did not formally assess inter-rater reliability.

Based on a tool developed by Black *et al.* ^(^ ^25^ ^)^, the market survey checklist examined: the price (reported as range of three prices), quality (good, medium, poor), promotions (yes, no), shelf placement (most prominent (at eye level), least prominent (bottom shelf), somewhat prominent (other)), store placement (easy, neither easy or hard, hard to find within the store) and the availability of nutrition information (none, front-of-pack, back-of-pack, ingredients list) for each of the foods. In order to assess market infrastructure, the following features were examined: access to potable water; toilets and electricity; refrigeration; protection from the outside environment; and whether the market appeared to be clean. For each of the markets examined, the main types of foods sold in the market were assessed by examining the shelf/physical space occupied by the different types of foods. The food processing categories defined by Poti *et al.* ^(^ ^26^ ^)^, which include unprocessed/minimally processed (e.g. fresh plain milk), basic processed (e.g. unsweetened fruit juice), moderately processed (e.g. sweetened canned fruit) and highly processed (e.g. soda), were used to describe the overall types of foods included in the market. Although markets most often contained foods from each of these processing categories, enumerators made an assessment based on the majority of the foods available in the market. In addition, the overall degree of convenience of the foods available in the market (requires cooking, ready-to-heat, ready-to-eat) was assessed^(^ ^26^ ^)^.

### Consumer surveys

A total of 400 consumer surveys with men and women were completed at the same markets described above. Teams of two enumerators were stationed at the markets and asked all consumers over the age of 18 years to participate in the study. Intercept surveys were conducted outside the markets with consumers who were attending the market. Participants received a nominal incentive for participation (1500 Burmese Kyat; equivalent to approximately $US 1). Enumerators remained at the market until they had completed a total of twenty intercept surveys. We excluded thirty-eight surveys given that the respondents did not reside in our study settings (total *n* 362). Enumerators approached consumers entering and exiting the market, informed them of the study and asked for their consent to participate. The surveys took approximately 5–10 min to complete and included demographic information, typical food consumption as well as questions related to food preferences. The food consumption questions asked, in a typical week, how often the participant ate: fruit, vegetables, red meat, processed snacks, sugary drinks, energy drinks, fast foods, traditional Burmese street foods and other street foods. Participants responded by selecting one of the following options: ‘never or occasionally’, ‘2–3 times per week’, ‘4–6 times per week’, ‘once per day’ or ‘more than once per day’. The categories were collapsed into ‘once or more/d’, ‘2–6 times/week’ and ‘never or occasionally’ for the analyses. To capture food preferences, consumers were asked how much they like (i.e. ‘a lot’, ‘a little’ or ‘do not like’) the following foods and beverages: salty or sweet processed snacks, soda or other sugary drinks, energy drinks, fast foods, street foods, red meat, fruit and vegetables. The survey instrument was piloted in Yangon prior to use in our study communities.

### Analyses

Focus group data were open coded and thematic analysis was used to examine themes related to the components of the food environment as described by the High Level Panel of Experts’ report^(^ ^5^ ^)^, in addition to consumer behaviour and diets, nutrition and health, using the qualitative data analysis software NVivo version 11.4.2.

Descriptive statistics were used to describe the composition of markets, food consumption patterns and food preferences based on the market and consumer surveys. The *χ* ^2^ test was used to examine differences between categorical variables. A *P* value of ≤0·05 was used to denote statistical significance. All quantitative analyses were conducted using the statistical software package IBM SPSS Statistics version 24. This study was approved by the Johns Hopkins University institutional review board. All study participants provided informed oral consent to participate in the study.

## Results

Results are organized based on the components of the food environment as defined by the High Level Panel of Experts’ report^(^ ^5^ ^)^: food availability and physical access; economic access; promotion, advertising and information; and food quality and safety. Findings from the focus group discussions, market and consumer surveys have been combined and described below. A breakdown of the demographic characteristics of focus group and consumer survey participants can be found in the online supplementary material, Supplemental Tables 1 and 2, respectively.

### Overview of food environment

The online supplementary material, Supplemental Fig. 1, depicts the maps of the food environments in each of the study locations developed as part of the participatory social mapping focus group exercise. All four food environments were dominated by the informal food sector where stalls separately selling vegetables, fruit, oil, fish and seafood, meat and other grocery items were prevalent along with street vendors and mobile carts. In the upper-income township of Yangon, both informal (e.g. street vendors) and formal food outlets (e.g. chain restaurants) were present. Although the rural village was limited in terms of the in-village stores, the nearby town (10 km from the village) offered a much greater variety of stores similar to those in the other urban areas examined.

**Fig. 1:**
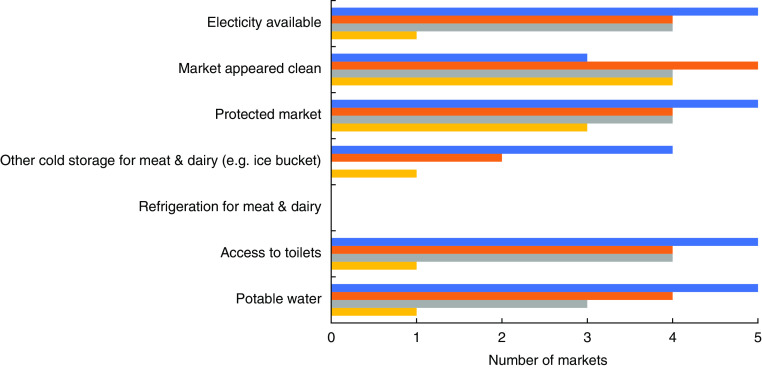
(colour online) An overview of the infrastructure in the markets in the food environments examined in each of the study settings (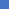, Yangon, upper income, urban; 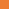, Yangon, lower income, urban; 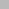, Magway, lower income, rural; 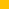, Dawei, middle income, coastal) in Myanmar, June–August 2017

Based on the market surveys, the degree of market infrastructure was quite variable across the twenty markets examined, particularly regarding access to toilets, potable water and electricity, with the markets in the coastal area of Dawei having the least infrastructure (Fig. 1). There was a lack of refrigeration for meat and dairy in all markets examined; however, the markets in Yangon were more likely to have access to other means of cold storage such as an icebox for fresh meat and dairy than those in the rural and coastal areas (*P* = 0·019).

### Food availability and physical access

**Fig. 2:**
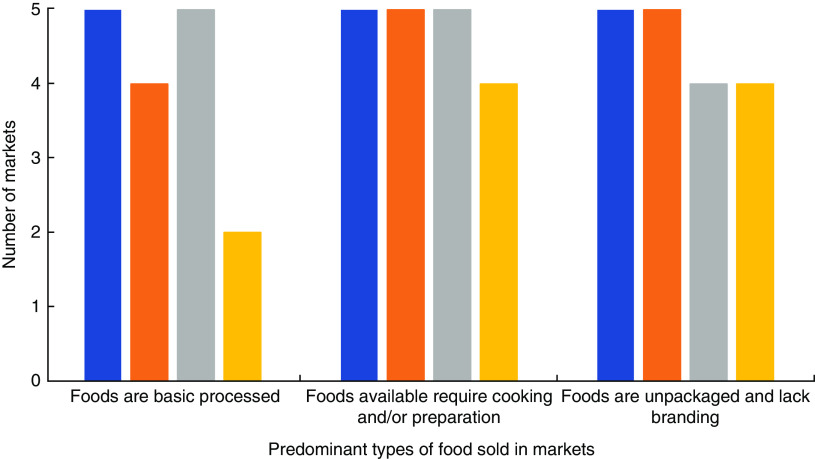
(colour online) An overview of the main types of food products in the markets in each of the study settings (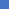, Yangon, upper income, urban; 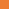, Yangon, lower income, urban; 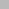, Magway, lower income, rural; 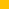, Dawei, middle income, coastal) in Myanmar, June–August 2017

Figure 2 provides an overview of the main types of foods available in the markets examined. The products available in each of the study locations were similar, with no statistically significant differences among the different study settings. However, there was a trend towards differences in the degree of processing of the foods sold in the markets based on study setting (*P* = 0·058). More specifically, there were fewer foods that were considered basic processed foods in Dawei compared with the other settings. Overall, the majority of foods available in the markets were minimally processed, unpackaged, lacked branding, and required cooking and/or preparation.

Although the market surveys illustrated that most of the foods available tended to be minimally processed, participants in the focus group discussions indicated changes in the availability of foods over time, including in relation to highly processed foods. One focus group participant stated that ‘foods are easily accessible and widely available now’ (participant 8, Dawei) from a variety of different food outlets. Although many foods were available, focus group participants perceived the lack of availability of healthy foods as a barrier to consuming them: ‘The main constraint is I can’t have easy access to health[y] and nutritious food’ (participant 4, Magway). At the same time, participants indicated an increased availability of health-oriented processed foods such as biscuits marketed to people with diabetes.

Physical proximity did not seem to be a barrier to accessing either highly processed and fried foods or fresh foods in the food environments examined in the present study. Even in the rural village in Magway, participants said that they had access to a wide range of foods through various mechanisms, including in-village stores and mobile vendors, the latter of which come to the village every morning selling fresh vegetables and meat. These vendors could provide foods that community members asked for and deemed a ‘reasonable price’. Moreover, although there were no fried chicken shops in the rural village, one focus group participant said: ‘If I want to eat some modern and palatable food which is not available in our village like fried chicken, Shan noddle or fried noodle, I go and buy them at the town market’ (participant 7, Magway). The borders of the food environment were porous with the food environment not only reflecting the foods that were available in a given township or village but beyond.

### Economic access

Although availability of foods, for the most part, was not considered a barrier to accessing the foods that consumers wanted to purchase, economic affordability was, particularly for the focus group participants from Magway, who were most likely to mention lack of income as a constraint: ‘What I need is money only to purchase food. Everything is available in the market’ (participant 1, Magway). In addition to the affordability of foods, a barrier to consuming healthier or more nutritious foods was the trade-off between the quality and price of foods over time. As a participant from Magway voiced in reference to edible oils: ‘In the past, price is high but we get good quality. But now, price becomes cheap and the quality becomes poor. So, we can buy a large quantity at a poor quality’ (participant 2). This meant that they could purchase and consume more oil but that they were not getting the quality they had once been able to receive. This was mainly attributed to palm oil being ubiquitous in the food supply. There was also a perception that food prices had changed (increased or decreased) over time depending on type of food; however, there was little consensus among focus group participants. Vegetables were considered affordable by nearly all the focus group participants, and meat and fish were considered less affordable among the lower-income focus group participants. Overall, the prices of highly processed foods such as instant noodles and potato chips were relatively cheap compared with fresh foods and, except for the rural markets in Magway, they were available in most of the markets surveyed.

### Promotion, advertising and information

Although the market surveys revealed that most foods available in the markets were unpackaged and unbranded foods, focus group participants indicated that the promotion and advertising of foods had increased over time. In particular, packaging had become more ‘pretty’/‘attractive’, persuading consumers to purchase snack foods for themselves and their children. Several focus group participants reported that they were purchasing processed snacks, mostly from small grocers and supermarkets, based on the packaging: ‘If it is well-packed, we are encouraged to buy it’ (participant 7, lower-income Yangon). Nevertheless, based on the market surveys the majority of foods available in the food environment still lack formal packaging.

Table 1 provides an overview of the display, promotion, placement, nutrition information and price of selected foods based on the market surveys. There was very little promotion of foods within the markets examined, with only pork being promoted in the markets in Dawei. There was more variability in the in-store display of foods as well as their quality. Although some packaged foods had the ingredients list included on the packaging, nutrition labelling was limited even for highly processed foods.

**Table 1:** The promotion, display, nutrition information and price of selected foods in markets across the study settings in Myanmar, June–August 2017†

Food group	Food	Geographic location	Market displayed promotions for the food item (no. of markets)	Item was displayed prominently on the shelf (no. of markets)	Store placement: food item was easy to find in the store (no. of markets)	Good quality of food item (no. of markets)	Nutrition information: ingredients list is present (no. of markets)	Price range (kyat)‡
Fruit	Banana	Yangon, upper income, urban	0 of 5	3 of 5	3 of 5	4 of 5	NA	1300–1500 per bunch
		Yangon, lower income, urban	0 of 5	5 of 5	4 of 5	5 of 5	NA	1200–1500 per bunch
		Magway, lower income, rural	4 of 4	4 of 4	3 of 4	0 of 4	NA	500–1200 per bunch
		Dawei, middle income, coastal	0 of 5	4 of 5	4 of 5	5 of 5	NA	500–1500 per bunch
	Papaya	Yangon, upper income, urban	0 of 5	5 of 5	2 of 5	4 of 5	NA	350–500 per piece
		Yangon, lower income, urban	0 of 3	2 of 3	1 of 3	2 of 3	NA	300–1500 per piece
		Magway, lower income, rural	0 of 4	3 of 4	1 of 4	4 of 4	NA	350–500 per piece
		Dawei, middle income, coastal	0 of 2	1 of 2	1 of 2	2 of 2	NA	500–1000 per piece
	Grapes	Yangon, upper income, urban	0 of 5	5 of 5	1 of 5	5 of 5	NA	2000–3000 per viss§
		Yangon, lower income, urban	0 of 5	3 of 5	1 of 5	3 of 5	NA	400–1000 per viss
		Magway, lower income, rural	0 of 4	3 of 4	2 of 4	4 of 4	NA	2000–3000 per viss
		Dawei, middle income, coastal	0 of 5	5 of 5	4 of 5	5 of 5	NA	5000–7000 per viss
Vegetables	Carrots	Yangon, upper income, urban	0 of 5	4 of 5	4 of 5	5 of 5	NA	1800–3000 per tical║
		Yangon, lower income, urban	0 of 5	3 of 5	2 of 5	3 of 5	NA	2000–3000 per tical
		Magway, lower income, rural	0 of 5	4 of 5	3 of 5	5 of 5	NA	1600–3000 per tical
		Dawei, middle income, coastal	0 of 4	3 of 4	3 of 4	4 of 4	NA	1500–2500 per tical
	Eggplant	Yangon, upper income, urban	0 of 5	4 of 5	3 of 5	5 of 5	NA	100–200 per piece
		Yangon, lower income, urban	0 of 5	3 of 5	2 of 5	4 of 5	NA	100–200 per piece
		Magway, lower income, rural	0 of 5	5 of 5	5 of 5	5 of 5	NA	50–100 per piece
		Dawei, middle income, coastal	0 of 4	4 of 4	4 of 4	4 of 4	NA	150–300 per piece
	Tomato	Yangon, upper income, urban	0 of 5	4 of 5	3 of 5	5 of 5	NA	1000–3000 per piece
		Yangon, lower income, urban	0 of 5	4 of 5	4 of 5	3 of 5	NA	1000–1500 per piece
		Magway, lower income, rural	0 of 5	5 of 5	5 of 5	5 of 5	NA	600–1000 per piece
		Dawei, middle income, coastal	0 of 5	5 of 5	5 of 5	5 of 5	NA	1000–1700 per piece
	Cabbage	Yangon, upper income, urban	0 of 5	4 of 5	3 of 5	5 of 5	NA	500–800 per piece
		Yangon, lower income, urban	0 of 5	3 of 5	4 of 5	4 of 5	NA	400–600 per piece
		Magway, lower income, rural	0 of 5	5 of 5	5 of 5	5 of 5	NA	600–800 per piece
		Dawei, middle income, coastal	0 of 5	5 of 5	5 of 5	5 of 5	NA	700–800 per piece
Red meat	Beef	Yangon, upper income, urban	0 of 5	4 of 5	3 of 5	5 of 5	NA	9000–10 000 per viss
		Yangon, lower income, urban	0 of 5	3 of 5	3 of 5	4 of 5	NA	8000–10 000 per viss
		Magway, lower income, rural	0 of 5	5 of 5	5 of 5	4 of 5	NA	10 000–11 000 per viss
		Dawei, middle income, coastal	0 of 3	3 of 3	2 of 3	1 of 3	NA	10 000 per viss
	Pork	Yangon, upper income, urban	0 of 5	5 of 5	4 of 5	4 of 5	NA	8000–9000 per viss
		Yangon, lower income, urban	0 of 5	4 of 5	3 of 5	4 of 5	NA	7000–9000 per viss
		Magway, lower income, rural	0 of 5	5 of 5	5 of 5	5 of 5	NA	8800–9800 per viss
		Dawei, middle income, coastal	5 of 5	5 of 5	1 of 5	2 of 5	NA	8000–10 000 per viss
	Mutton	Yangon, upper income, urban	0 of 5	4 of 5	3 of 5	4 of 5	NA	16 000–17 000 per viss
		Yangon, lower income, urban	0 of 4	2 of 4	2 of 4	3 of 4	NA	15 000–17 000 per viss
		Magway, lower income, rural	0 of 5	5 of 5	5 of 5	4 of 5	NA	11 000–12 000 per viss
		Dawei, middle income, coastal	0 of 2	2 of 2	2 of 2	2 of 2	NA	10 000–15 000 per viss
Processed Snacks	Potato chips	Yangon, upper income, urban	0 of 4	3 of 4	4 of 4	4 of 4	2 of 4	100 per bag
		Yangon, lower income, urban	0 of 4	2 of 4	2 of 4	2 of 4	3 of 4	100–200 per bag
		Magway, lower income, rural	NA	NA	NA	NA	NA	NA
		Dawei, middle income, coastal	0 of 2	0 of 2	0 of 2	2 of 2	1 of 2	200 per bag
	Instant noodles	Yangon, upper income, urban	0 of 5	3 of 5	3 of 5	4 of 5	5 of 5	150 per bag
		Yangon, lower income, urban	0 of 5	3 of 5	2 of 5	2 of 5	5 of 5	150–200 per bag
		Magway, lower income, rural	0 of 5	3 of 5	3 of 5	5 of 5	4 of 5	150–200 per bag
		Dawei, middle income, coastal	0 of 5	5 of 5	0 of 5	4 of 5	5 of 5	150–200 per bag
Sugary drinks	Local cola brand (Max Plus)	Yangon, upper income, urban	0 of 5	3 of 5	5 of 5	5 of 5	5 of 5	350–400 per 300 ml
		Yangon, lower income, urban	0 of 5	1 of 5	2 of 5	3 of 5	5 of 5	300–450 per 300 ml
		Magway, lower income, rural	0 of 5	1 of 5	2 of 5	5 of 5	4 of 5	400–450 per 300 ml
		Dawei, middle income, coastal	0 of 4	1 of 4	1 of 4	4 of 4	4 of 4	450–500 per 300 ml
Energy drinks	Local brand (Burn)	Yangon, upper income, urban	0 of 5	3 of 5	5 of 5	5 of 5	5 of 5	450–500 per 300 ml
		Yangon, lower income, urban	0 of 5	2 of 5	3 of 5	4 of 5	5 of 5	350–500 per 300 ml
		Magway, lower income, rural	0 of 4	4 of 4	2 of 4	4 of 4	4 of 4	450–500 per 300 ml
		Dawei, middle income, coastal	0 of 5	3 of 5	2 of 5	5 of 5	5 of 5	350–500 per 300 ml

The numbers reflect the number of markets examined. In some cases, the item was not available (NA) in a given market. In these cases, only the markets that contained the product were examined.

† The information captured in this table is adapted from a checklist developed by Black *et al.* ^(^ ^25^ ^)^.

‡ 1 $US is equivalent to 1350 Burmese Kyat.

§ Viss is a local unit of measurement that equates to 1·63 kg.

║ Tical is a local unit of measurement that equates to 16·32 g.

Focus group participants observed shifts in the way in which foods were displayed in stores over time, making the variety of food available more visible. In terms of the promotion of foods, none of the focus group participants mentioned mass media advertisements; however, free samples of instant noodles, instant coffee and bouillon cubes were distributed in shops in urban areas to promote their purchase. Two focus group participants reported that door-to-door sales were used in villages: ‘There are female sales promoters who advertise and explain the quality of their products. Sometimes they go door-to-door and advertise around the village’ (participant 3, Yangon, upper income). When asked about the nutritional information available on the labels of packaged foods, very few focus group participants observed nutrition labelling. However, a few participants mentioned a ‘FDA recommended’ label which was perceived to be an indication of improved quality.

### Food safety and quality

There were three main changes to the quality and safety of foods over time that were noted by focus group participants: (i) the physical appearance and organoleptic (i.e. mouthfeel, texture, etc.) properties of food were changing; (ii) the adulteration of food had increased; and (iii) deterioration in the taste of food over time. As one focus group participant stated: ‘You can see cabbages are bigger than normal size. But chemicals have been added to them’ (participant 6, Dawei). In terms of the adulteration of foods, participants perceived an increase in use of formalin in baked goods, fish and some vegetables, fertilizer in fish paste to help preserve the food and extend its shelf-life, as well as an increase unsafe use of pesticides. As one participant from Dawei stated: ‘For me, chemicals are added to more and more foods. So, most of the foods are not healthy’ (participant 2). Focus group participants stated that they were reluctant to eat fruit based on the perceived chemical content and would prefer to purchase organic food; however, both availability and price were seen as barriers to its purchase. In addition to the use of chemicals, focus group participants indicated that many of the oils that were available in the food environment were mislabelled, with higher-quality oils (e.g. sesame or groundnut) often being mixed with palm oil but labelled as the higher-quality oil. Focus group participants in all four study locations showed strong concern and condemnation for the way foods were now being produced. As a participant from a high-income township in Yangon stated: ‘People do not have ethics in the food production process’ (participant 2).

### Consumer behaviour

#### Food consumption patterns

**Fig. 3:**
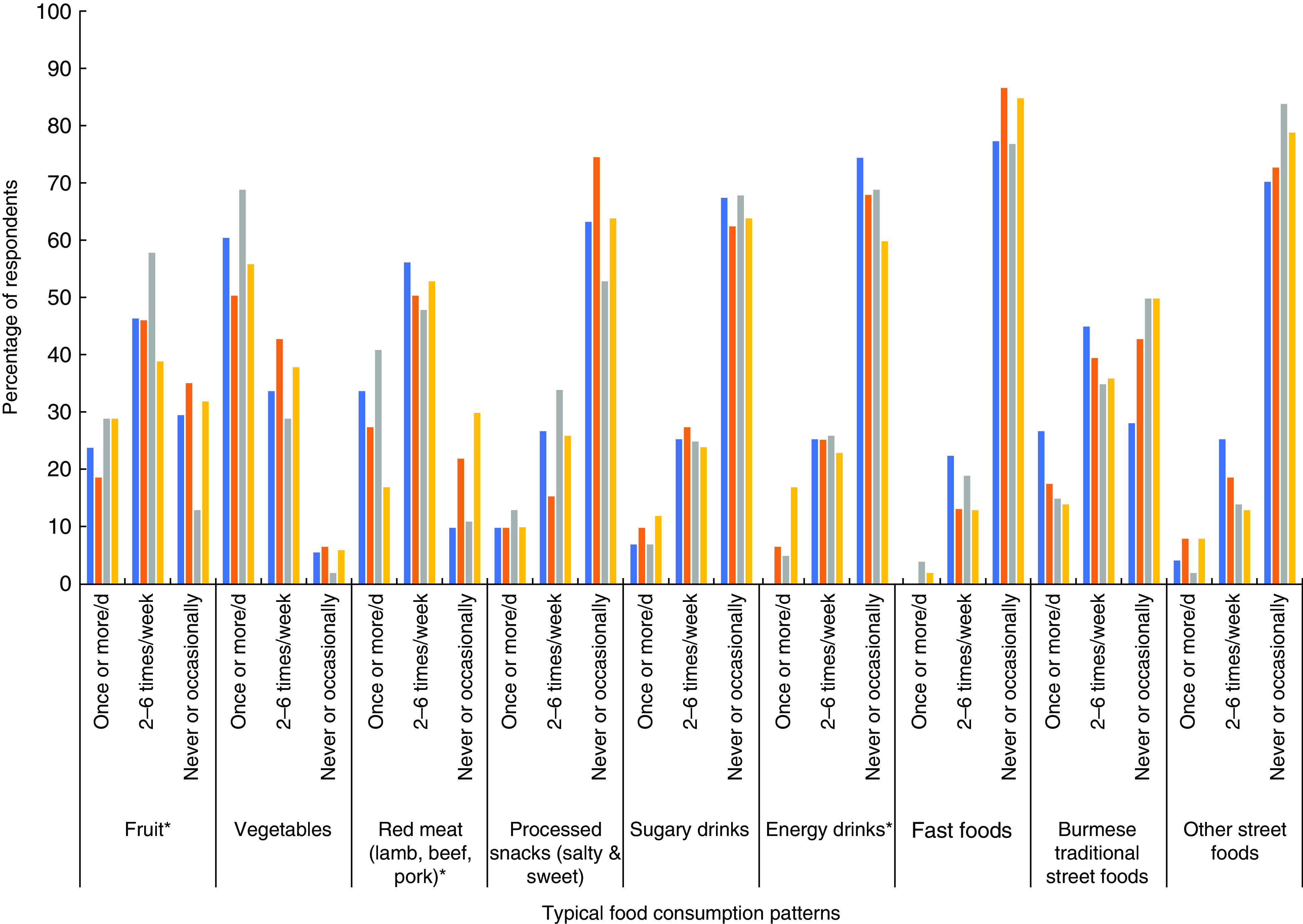
(colour online) An overview of consumers’ typical intakes across the different study settings (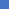, Yangon, upper income, urban; 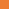, Yangon, lower income, urban; 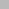, Magway, lower income, rural; 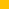, Dawei, middle income, coastal) in Myanmar, June–August 2017. *Statistically significant at *P* ≤ 0·05 (*χ* ^2^ test)

**Fig. 4:**
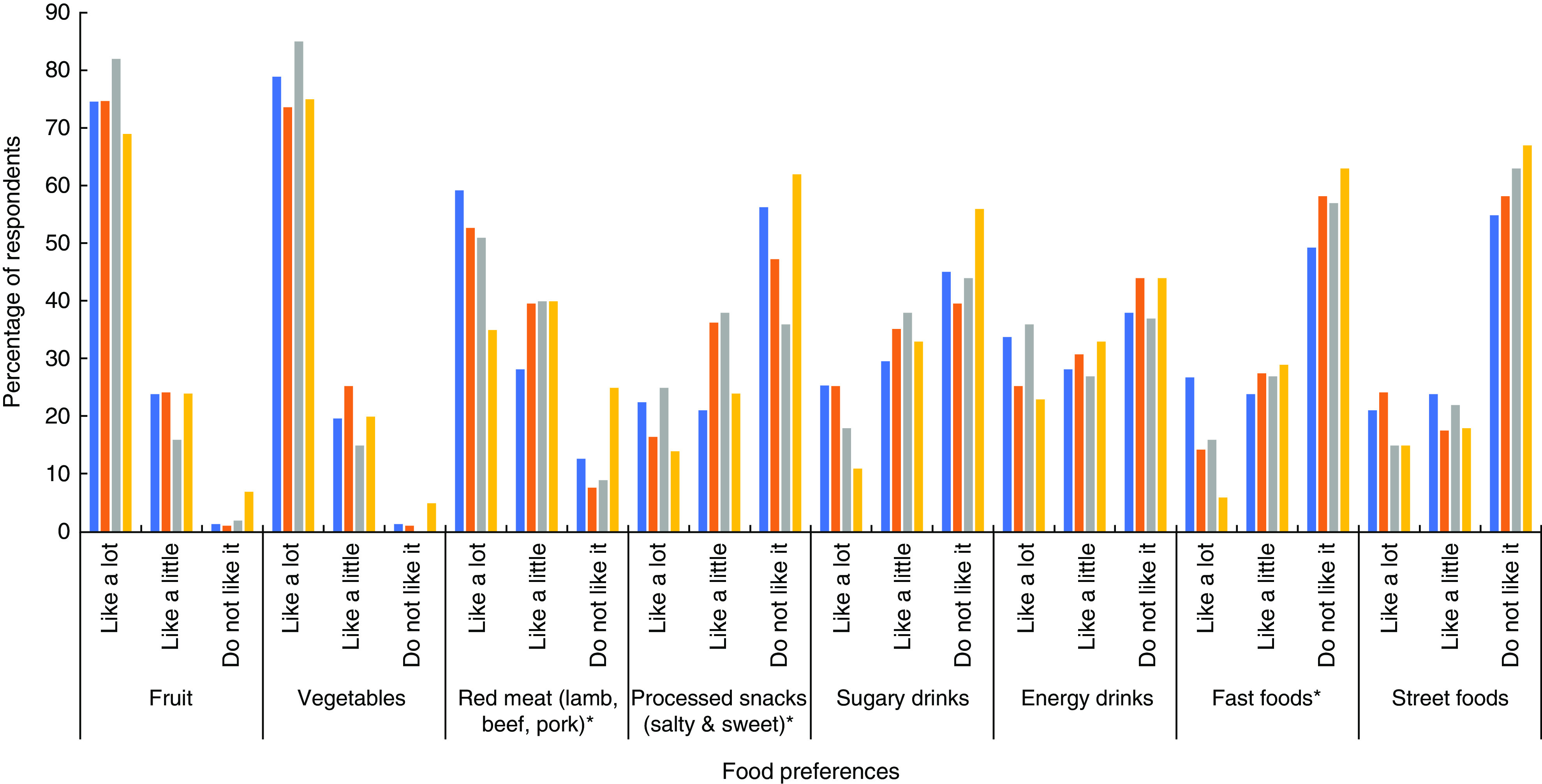
(colour online) Consumers’ reported food preferences across the different study settings (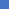, Yangon, upper income, urban; 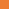, Yangon, lower income, urban; 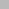, Magway, lower income, rural; 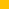, Dawei, middle income, coastal) in Myanmar, June–August 2017. *Statistically significant at *P* ≤ 0·05 (*χ* ^2^ test)

Based on the consumer survey findings, Fig. 3 provides an overview of the typical food consumption patterns in the different study settings. Overall, the reported food consumption patterns were similar across study settings. Over half of survey participants reported consuming vegetables once or more daily, compared with less than a third consuming fruit once or more daily. Red meat consumption was high among the survey participants, with significant differences across study settings (*P* <0·001). The highest consumption levels were reported in the rural area of Magway, while the lowest levels were in the coastal region of Dawei, where food preference for red meat was relatively low (25 % reported not liking red meat compared with 7–12 % in the other settings; Fig. 4). Based on our focus group data, Dawei was experiencing an H5N1 influenza outbreak during the time of data collection which may have also influenced meat consumption, given that some focus group members mentioned avoiding pork for this reason. Across all study locations, the majority of participants reported never or only occasionally eating sugary drinks, energy drinks or fast foods. However, traditional Burmese street foods were consumed frequently. We also examined differences in consumption patterns based on sex and education within each of the study settings (data not shown). There was a significant difference in reported meat consumption across education levels (*P* = 0·039) in the upper-income participants in Yangon as well as in the coastal area of Dawei (*P* = 0·042). There were also differences in the consumption of street foods as well as salty or sweet processed snack foods by age and sex in some of the study settings.

### Food preferences

Figure 4 provides insight into the food preferences of participants in the different study settings based on the consumer surveys. Overall, there were significant differences in preferences for processed snack foods, fast foods and red meat across the different study settings. In many cases, the preferences for processed foods appeared to be higher in the urban settings compared with the coastal setting in particular. In the rural setting, preferences for some processed food and beverage items, such as snack foods and energy drinks, were quite high. Study participants in all regions reported a preference for both fruits and vegetables. There were no differences in food preferences based on sex or education in the different study settings, except for fruit preference being associated with education in the upper-income Yangon setting. More specifically, 33 % of those with less than primary school compared with 77 % of college educated participants reported liking fruit a lot (*P* = 0·001).

Although the consumer survey data suggested that the majority of consumers were not eating highly processed and fast foods frequently, and there was variability in terms of preferences for these foods, focus group participants indicated a growing preference and increasing consumption of these foods. As one participant from Dawei stated: ‘I usually consume deep-fried food’ (participant 3). Moreover, many of the focus group participants mentioned that they would prepare fried snack foods such as fried chicken and potato chips in their own home: ‘If I want to eat potato chips, I fry them at my home’ (participant 2, Magway). Although focus group participants indicated that they preferred snack foods, these were not solely imported snack foods but also Burmese fried and processed snacks. Some of the focus group participants also noted the influence their families’ food preferences have on the foods they purchase. In particular, several participants noted that their children had a preference for processed snack foods which would lead them to purchase them: ‘For my kids, I buy potato chips and biscuits at convenience store near my house’ (participant 4, Dawei).

### Diets and nutrition and health outcomes

Focus group participants associated health with food safety. Although they recognized that the changes in the food supply had led to increases in disease prevalence, including diabetes and hypertension, these increases were mainly attributed to food safety concerns rather than concerns related to the consumption of foods that may be associated with increased risk of diet-related non-communicable diseases. However, as a participant from a higher-income township in Yangon stated: ‘We are buying the diseases with our money’ (participant 2).

In general, focus group participants had limited knowledge related to what constitutes a healthy diet. Many thought that fried chicken and potato chips had a high nutritional value, while some leafy greens and canned fish did not. Nearly all focus group participants indicated that instant noodles provided little nutritional value. Despite limited knowledge in terms of the nutritional quality of some foods, some of the middle- and upper-income focus group participants indicated making changes to their diets based on health concerns. More specifically, some participants mentioned reducing monosodium glutamate and oil consumption, consuming specific oils to promote health and consuming healthy processed foods (e.g. biscuits for people with diabetes). Nevertheless, there seemed to be some discrepancies between what was perceived to be healthy and what might be recommended for health. For example, two focus group participants mentioned replacing monosodium glutamate with sugar to improve the healthiness of their curries.

## Discussion

The present study describes the food environment in four different settings in Myanmar. Using a combination of quantitative and qualitative methods, we found that the variety of foods available in the Burmese food supply has been increasing over time but that quality has reportedly diminished. Although processed food availability has increased, intakes and preferences for these foods were quite variable across study settings. Health was seen primarily through a food safety lens, which has important implications for food choices. Lastly, physical proximity was not seen as barrier to accessing most foods.

As with other countries undergoing the nutrition transition, we found that the variety of food available in the food supply was increasing in Myanmar. However, coinciding with the increase in the variety of food, there was also a perceived reduction in its overall quality. Importantly, the reduction in quality was not seen primarily in terms of increases in salt, sugar and unhealthy fat, but more related to an increase in chemicals, contaminants, adulteration of foods as well as reductions in taste. This is an important consideration for conducting research examining food environments in LMIC contexts. In high-income countries, the food safety aspect of the food environment is often overlooked, but it remains a serious issue in LMIC such as Myanmar. Moreover, the way in which diets are perceived in terms of health can be quite different from one country the next. This points to the need to ensure that research examining health aspects of food environments in LMIC includes information related to consumers’ perceptions of what constitutes a healthy diet and the reasoning for interpreting health in that way.

### Food safety risks

Although systematic surveillance of the food safety risks in the Burmese food supply does not currently exist, there has been an increasing number of crackdowns by the Food and Drug Administration (FDA) of Myanmar on food safety threats^(^ ^27^ ^)^. Addressing food safety risks in the country is considered a priority area^(^ ^28^ ^)^. These risks include serious food poisoning outbreaks due to microbiological contamination, improper use of additives and the presence of other adulterants and environmental contaminants, among others^(^ ^28^ ^)^. However, given the predominantly informal food sector in Myanmar it will be difficult to address all the food safety concerns without marked increases in FDA capacity^(^ ^27^ ^)^. Without greater transparency in the quality of foods in the food supply, including those that are imported from surrounding countries, it is likely that the mistrust will prevail.

Some of the foods (e.g. leafy greens, fruits and vegetables) that consumers reported being wary of in the focus groups due to the overuse of pesticides were foods that are considered nutritious. Although our consumption data suggested that vegetables were still consumed by most consumer survey participants once or more daily, the same was not true of fruit where about a third of participants never or occasionally consumed fruit. Food safety concerns have been identified as a problem in many developing countries, given the weaknesses in technical infrastructure, resources, regulatory frameworks and enforcement capacity^(^ ^29^ ^)^. There is also often a lack of knowledge related to the appropriate use of pesticides among farmers, including in Myanmar^(^ ^30^ ^)^. Without investment in these areas, it is likely that consumers will continue to refrain from consuming some nutrient-rich foods due to food safety concerns.

### The need for increased nutrition-related knowledge

We found there was a recognition that alongside the nutrition transition, an epidemiological transition was underway. Although there was acknowledgement that the prevalence of diet-related disease, including diabetes and cancers, was increasing, this was largely attributed to the food safety concerns in the food supply rather than increased consumption of foods high in sugar, salt and unhealthy fat. There is a need to increase knowledge related to what constitutes a healthy diet and the relationship between food and diet-related non-communicable diseases in Myanmar. One of the challenges in terms of communicating this relationship is the lack of nutrition information available for most foods. Although the ingredients lists were available for the majority of packaged foods, nutrition labelling was not. Easy-to-interpret front-of-pack labelling can help to inform improved food choices^(^ ^31^ ^)^ and has the potential to benefit consumers in Myanmar.

### Porous food environment borders

Much of the food environment literature in high-income countries has focused on physical proximity of foods as an indicator of which foods a given person or household is able to access^(^ ^7^ ^)^. We found that physical proximity was not deemed a major barrier to accessing foods, yet the affordability of foods was considered a barrier. This finding is similar to the Korean context where financial and socio-behavioural rather than geographical barriers were seen to be more critical in terms of influencing food choice^(^ ^32^ ^)^. In our study, even if participants did not have access to highly processed and fried foods in their surrounding food environment they were preparing these foods themselves or accessing them from nearby towns. Physical boundaries of food environments were fluid, with mobile vendors selling foods, including those that are fresh and minimally processed, in the rural villages on a daily basis. This has important implications for the way in which researchers examine food access within a given food environment. Given that the food environments extend beyond the limits of a given community this means that interventions aimed at addressing food purchasing and consumption need to go beyond the immediate surroundings as well.

### Food marketing

Trade liberalization has been associated with changes to the availability of foods in the food supply along with increases in food advertising and marketing^(^ ^33^ ^)^. Some of this has been attributed to the supermarket revolution in Asia and Africa^(^ ^34^ ^)^, although we found larger reliance on informal markets rather than supermarkets for food purchasing in Myanmar. The majority of the foods that were available in the informal markets examined as part of the present study were unpackaged, unlabelled and were not being promoted; however, focus group participants reported an increase in the sophistication of packaging of snack food and beverage products over time, mostly from the formal markets. We also found that although focus group participants were not reporting high amounts of mass media advertising, food marketing prevailed in other forms. The use of free samples and door-to-door sales is one way to expose consumers to new foods, many of which are highly processed. This same phenomenon has been observed in Brazil where snack foods targeted at low-income consumers were being sold door-to-door in the outskirts of several cities^(^ ^35^ ^)^. Food packaging aimed at enticing consumers to purchase foods appeared to influence food purchasing more than other forms of marketing. Focus group participants noted that much of this packaging seemed to target children. This is problematic given that children are more susceptible to misleading marketing^(^ ^36^ ^)^. Myanmar currently has no guidelines related to marketing to children. Global recommendations on the marketing of foods and beverages to children exist^(^ ^37^ ^)^, which can lay the foundation for national recommendations. In recent years, several countries have adopted legislation regarding the marketing of foods and beverages to children^(^ ^38^ ^)^. Adopting similar legislation could prove beneficial in Myanmar.

### Limitations

The current project is not without limitations. The main study limitations are our reliance on convenience samples for our consumer surveys, the relatively small number of focus groups and a lack of formal assessment of inter-rater reliability for the market surveys. Another limitation of the study was that we examined only four different food environments in Myanmar, and thus our results are not representative of all the food environments that exist across Myanmar as a whole. Additional research in different settings across the country is needed to better characterize the breadth of food environments that exist. Moreover, future research should put more emphasis on the role of socio-economic status as an important determinant of food preferences and consumption patterns, as well as a shaper (through demand) of food environments.

## Conclusion

Consumers are currently exposed to a larger variety of foods, of varying quality, in the food environments with which they interface in Myanmar. The influx of highly processed and fried foods seems to have reached both urban and rural areas; however, there appears to be variability in the reported intakes of these foods across different food environments within the country. In order to improve the quality of the food environments in Myanmar, improvements in food safety, nutrition labelling and restrictions on food marketing to children should be prioritized.

Interventions aimed at improving the quality of the food environments that consumers interface with need to be grounded in the contextual realities of those food environments. There is a dearth of literature that characterizes the food environments in LMIC and the current research contributes to filling this gap. Additional research is needed in other LMIC contexts to begin to better characterize the different types of food environments in these countries, their transition over time, and the role of interventions and policies aimed at improving the quality of the food environment. Adopting mixed-methods approaches, similar to that used in the present study, may be more appropriate in these contexts as compared with relying solely on more geographical-based (e.g. geographic information systems) methods, often used in high-income countries. Using mixed methods helps to provide a deeper picture of the reality of food environments in LMIC as well as provide insight into how consumers interface with them.

## Supplementary material

For supplementary material accompanying this paper visit https://doi.org/10.1017/S1368980018003427. click here to view supplementary material
