# Supplementary material for: The interface between consumers and their food environment in Myanmar: an exploratory mixed-methods study
Source: Public Health Nutr. 2018 Dec 18;22(6):1075–88. doi: 10.1017/S1368980018003427 (PMC6536824; doi:10.1017/S1368980018003427)
Supplement: Supplementary file 1 [file S1368980018003427sup001.docx]

**Supplementary material**

**Supplemental Table 1. Demographic characteristics of focus group participants in Myanmar**

|  |  | Yangon upper income (n=8) | Yangon lower income (n=8)_ | Magway (n=8) | Dawei  (n=8) |
| --- | --- | --- | --- | --- | --- |
| Highest level of education completed (n) | Less than primary school | 0 | 2 | 6 | 3 |
|  | Primary school | 0 | 2 | 0 | 1 |
|  | Secondary school | 4 | 0 | 0 | 0 |
|  | High school | 0 | 4 | 0 | 4 |
|  | College | 4 | 0 | 2 | 0 |
| Employment status (n) | Government employee | 3 | 0 | 0 | 1 |
|  | Employed by a non-government organization | 0 | 0 | 0 | 0 |
|  | Self-employed | 0 | 2 | 3 | 2 |
|  | Homemaker | 5 | 6 | 2 | 5 |
|  | Farm/casual worker | 0 | 0 | 3 | 0 |
| Age (years) | Mean (range) | 40 (21-55) | 39 (30-50) | 40 (24-57) | 42 (27-61) |
| Ethnic group (n) | Burmese | 7 | 8 | 8 | 0 |
|  | Chin | 1 | 0 | 0 | 0 |
|  | Indian | 0 | 0 | 0 | 0 |
|  | Dawei | 0 | 0 | 0 | 8 |

**Supplemental Table 2. Demographic characteristics of consumer survey participants in Myanmar**

|  |  | Yangon upper income (n=71) | Yangon lower income (n=91)_ | Magway (n=100) | Dawei  (n=100) | p-value |
| --- | --- | --- | --- | --- | --- | --- |
| % Female |  | 56% | 48% | 50% | 50% | .767 |
| Highest level of education completed | Less than primary school | 4% | 18% | 7% | 28% | <.001 |
|  | Primary school | 17% | 38% | 31% | 31% |  |
|  | Secondary school | 32% | 31% | 30% | 29% |  |
|  | High school | 10% | 4% | 7% | 2% |  |
|  | College | 37% | 9% | 25% | 10% |  |
| Employment status | Government employee | 1% | 3% | 8% | 5% | .277 |
|  | Employed by a non-government organization | 16% | 11% | 15% | 17% |  |
|  | Self-employed | 68% | 75% | 67% | 69% |  |
|  | Homemaker | 13% | 4% | 8% | 8% |  |
|  | Other | 2% | 7% | 2% | 1% |  |
| Age | Mean ± SD | 39 ± 12 | 41 ± 12 | 39 ± 13^a^ | 43 ± 11^b^ | .039 |
| Ethnic group | Burmese | 73% | 64% | 96% | 85% | <.001 |
|  | Karen | 6% | 18% | 0% | 1% |  |
|  | Indian | 16% | 6% | 2% | 2% |  |
|  | Other | 5% | 12% | 2% | 12% |  |

**Supplemental Fig. 1. Food environment maps from participatory social mapping in four food environments in Myanmar**


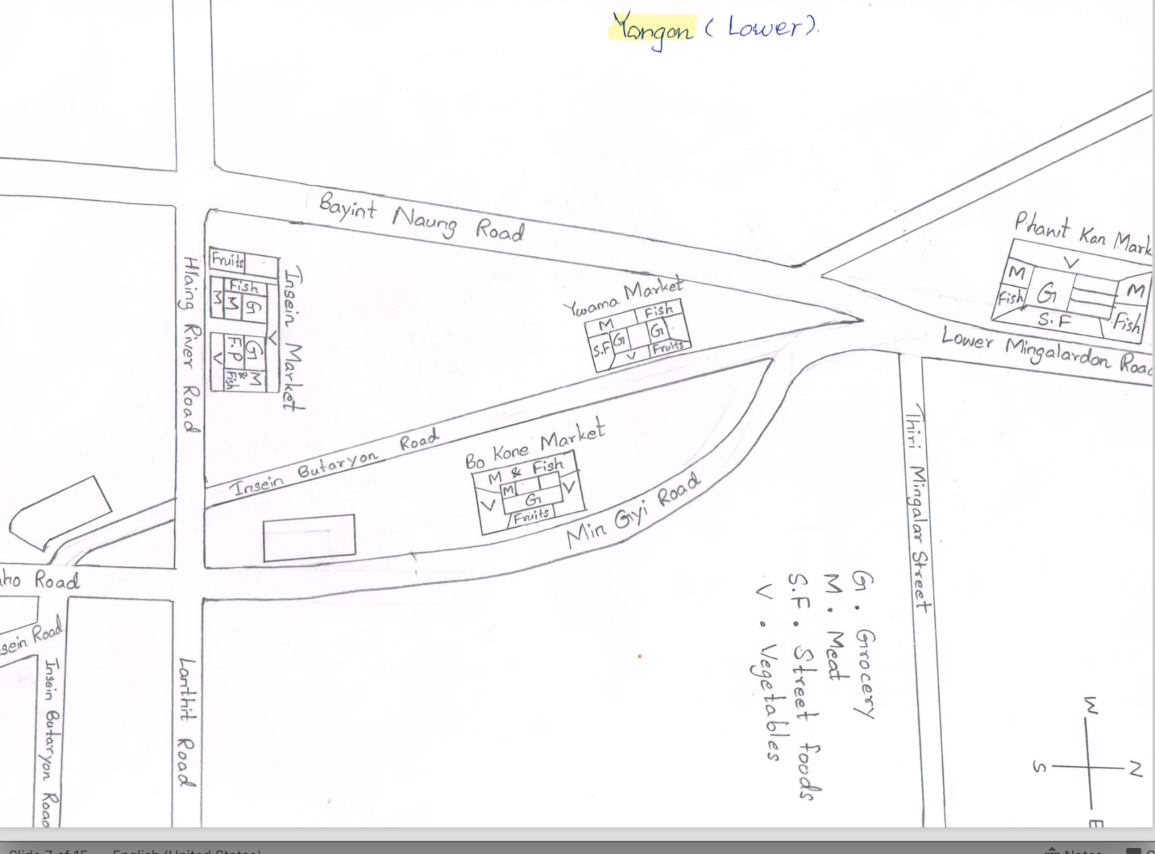


Yangon Lower Income (urban)


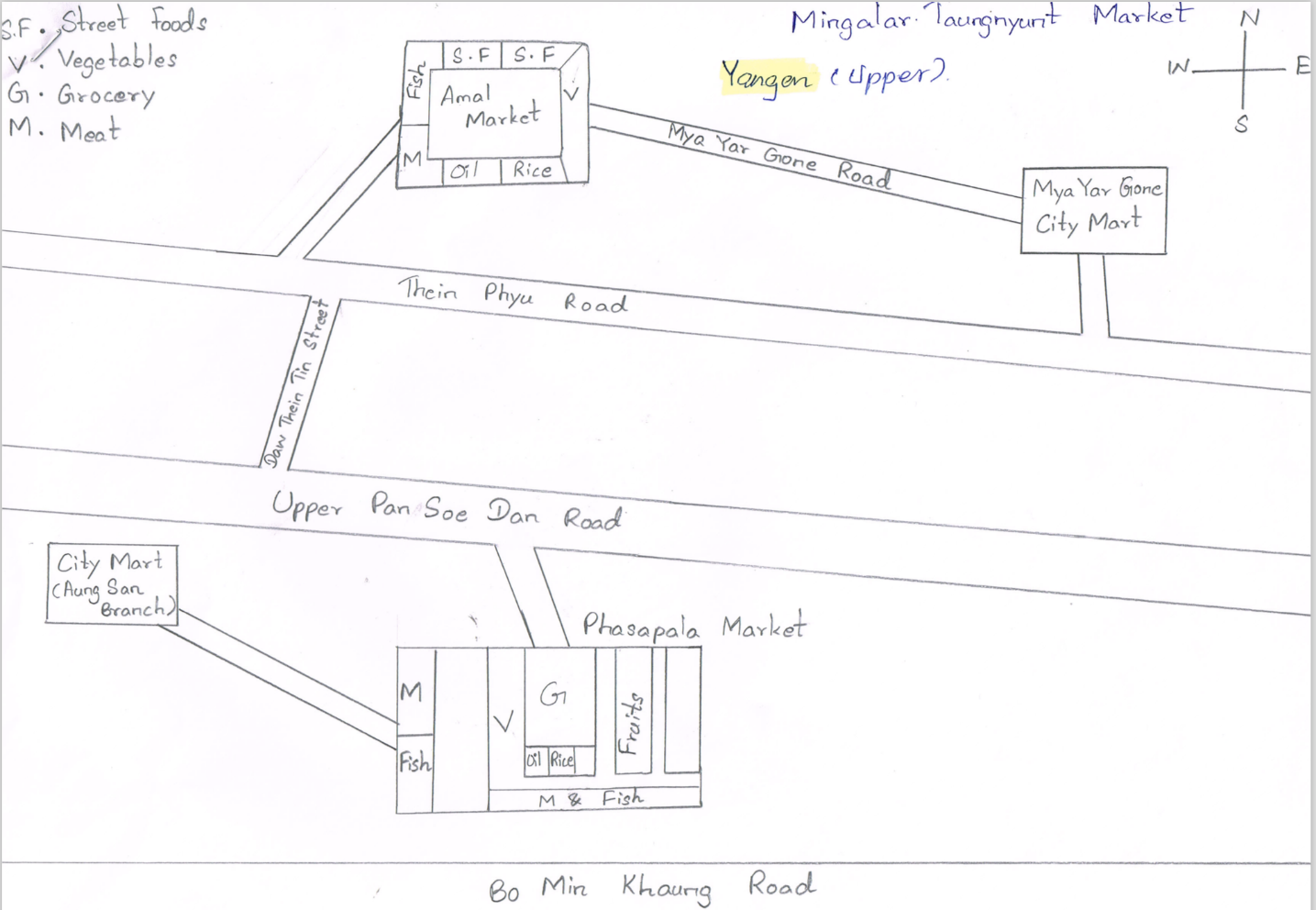


Yangon Upper Income (urban)


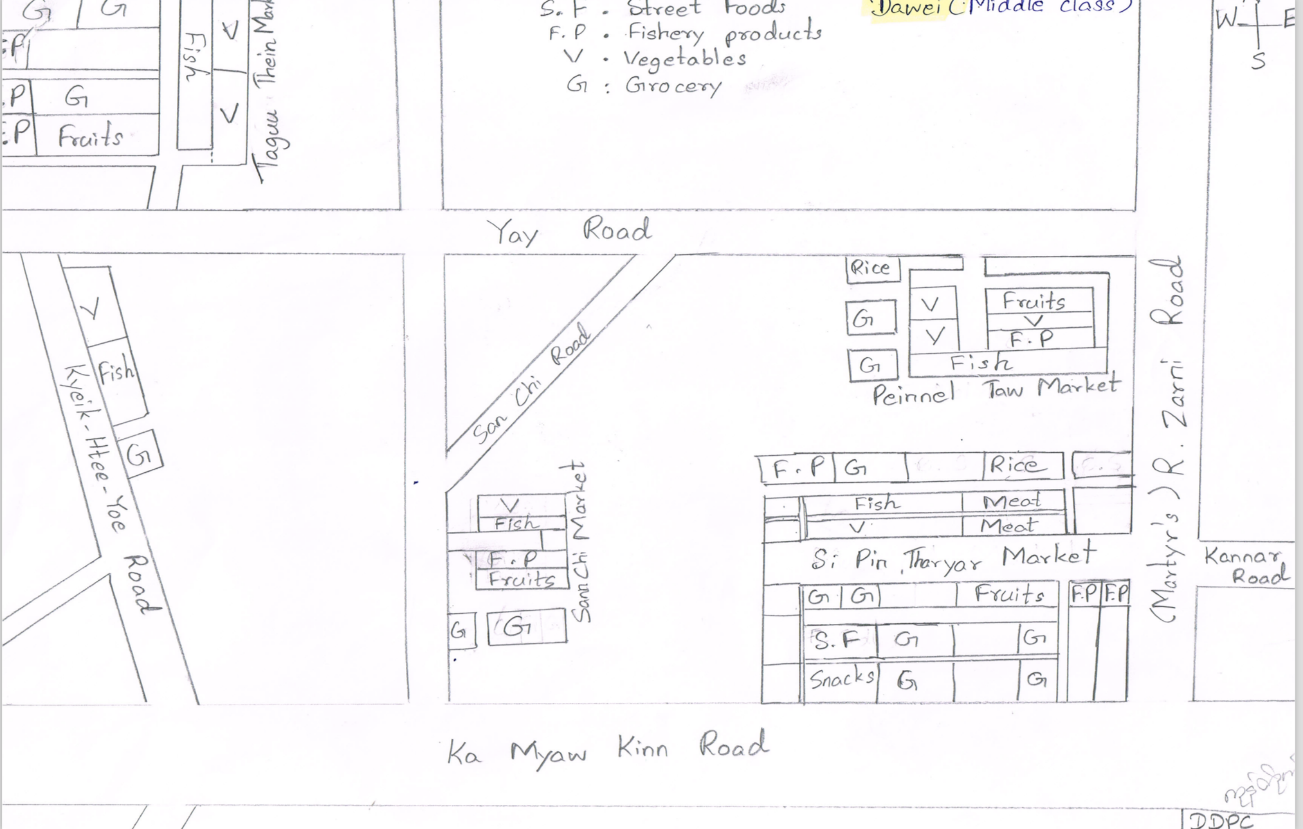

Middle Income Dawei (coastal)


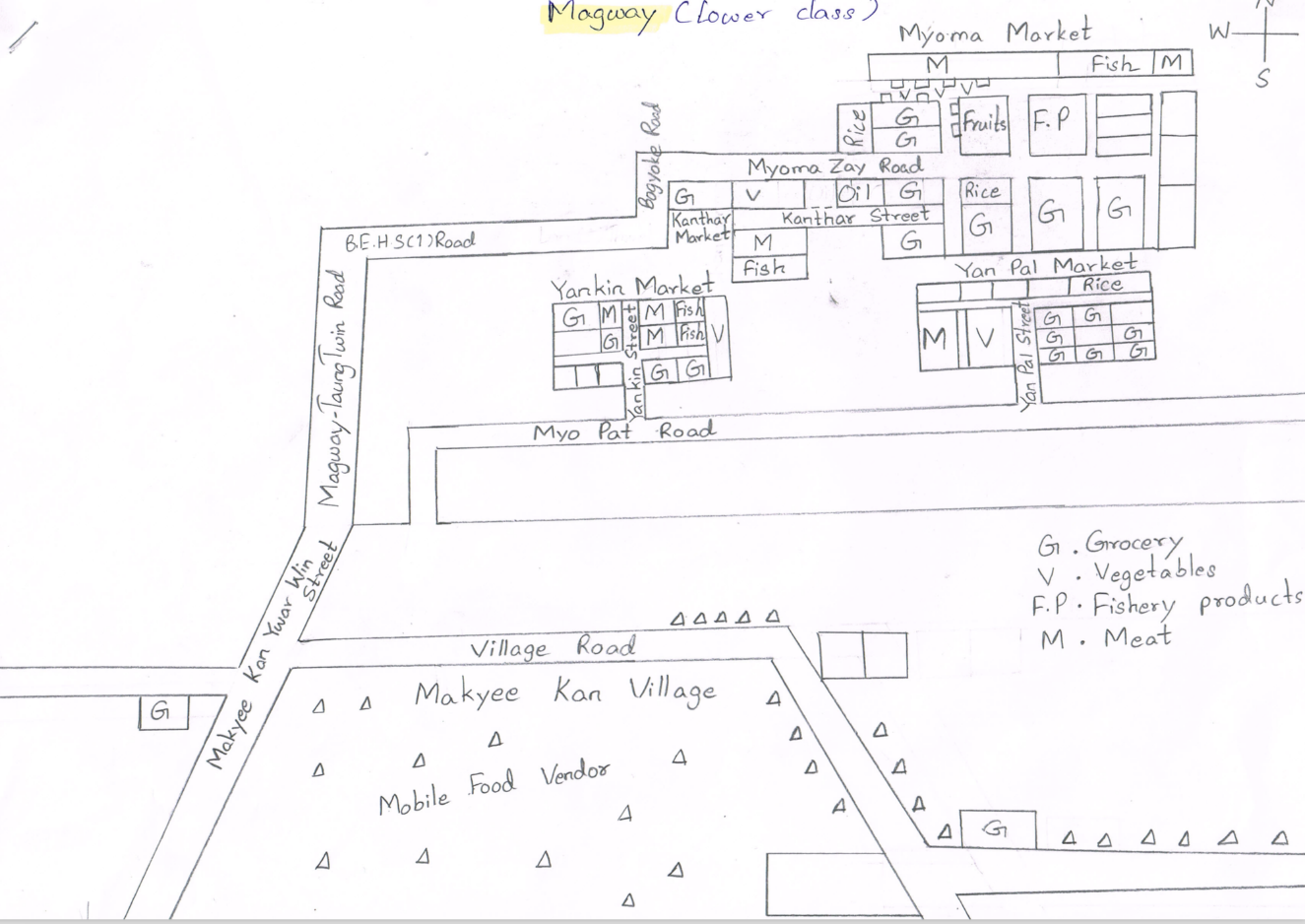


Lower Income Magway (rural)
